# Digital Assessment of Gingival Dimensions of Healthy Periodontium

**DOI:** 10.3390/jcm10081550

**Published:** 2021-04-07

**Authors:** Hyun-Chang Lim, Jaemin Lee, Dae-Young Kang, In-Woo Cho, Hyun-Seung Shin, Jung-Chul Park

**Affiliations:** 1 Department of Periodontology, Periodontal-Implant Clinical Research Institute, School of Dentistry, Kyung Hee University, Seoul 02447, Korea; Hyun-Chang.Lim@khu.ac.kr; 2 Department of Periodontology, College of Dentistry, Dankook University, Cheonan-si 31116, Korea; j39865@dent.dku.edu (J.L.); gd@dent.dku.edu (D.-Y.K.); sinuslift@hanmail.net (I.-W.C.); perioshin@dankook.ac.kr (H.-S.S.)

**Keywords:** attached gingiva, digital technology, keratinized gingiva, intraoral scanning

## Abstract

The aim of the present study was to re-visit the gingival dimension using digital scanning in a healthy Korean population. Forty-eight periodontally healthy volunteers (38 males and 10 females, mean age: 24.3 ± 2.2 years) were included. The mucogingival junction was highlighted using 2.5% diluted iodine solution. Then, the facial gingiva and mucosa of both jaws were digitally scanned using an intraoral digital scanner. Using computer software and periodontal probing, the heights and areas of keratinized gingiva (KG) and attached gingiva (AG) were measured. Similar distribution patterns in the gingival heights were noted in the maxilla and mandible. The maxilla showed substantially greater gingival values than the mandible. The heights of the KG and AG were notably smaller on the mandibular first premolar (2.37 mm and 1.07 mm, median value) and second molar (3.28 mm and 1.78 mm) than on the other teeth. The area of the KG was the largest in the canine (63.74 mm^2^ and 46.85 mm^2^) and first molar (64.14 mm^2^ and 58.82 mm^2^) in each jaw. Mandibular first and second molars, mandibular canine, and maxillary canine showed the highest value of the area under the receiver operation characteristics curve (>0.7) for differentiating between males and females. The gingival dimensions recorded using intraoral scanner demonstrated similar distribution patterns as in previous studies.

## 1. Introduction

Several studies have investigated the dimensions of the keratinized gingiva (KG) and attached gingiva (AG) around natural dentition [1,2,3,4], in pursuit of obtaining anatomic information and finding adequate gingival dimension for periodontal and mechanical stability. Especially, some surgical procedures, such as bone denudation procedure and apically positioned flap surgery, were once proposed for the latter purpose [5,6].

Literature demonstrated heterogeneity regarding the role of KG/AG. A representative study by Lang and Löe demonstrated that 2 mm of KG (1 mm of AG) was sufficient for periodontal health in the presence of professional maintenance [2]. On the contrary, others published later failed to show no specific relationship between KG/AG and periodontal health [3,7], leading to a consensus that there is no adequate gingival dimension for periodontal health. However, recent evidence suggests a substantial role of the gingival dimension as a part of periodontal phenotype to prevent gingival recession [8,9].

In terms of understanding the esthetics and pathophysiology of the periodontium and adjacent structures, dental clinicians must have an anatomical overview of the periodontium, such as dimension, tendency, and composition. From histological and morphological points of view, the KG and AG are differentiated. AG is firmly attached to the root cementum and alveolar bone by the insertion of collagen fibers, whereas KG is a union of a mobile gingival zone (outer surface of the bottom of the gingival sulcus or the periodontal pocket) and AG. However, clinical measurements of the height of the AG do not seem to be unequivocal because the measurement of the mobile zone is affected by many factors [10].

In order to assess the gingival dimensions, previous studies generally used a periodontal probe and a caliper [2,11,12,13]. However, these measurement methods may have limitations, which are derived from set graduations on measuring tools, interference of adjacent anatomic structures when measuring, condition of the periodontium, and expertness level. Current widespread digital technology in the dental field may provide a new opportunity to compensate the above limitations. A recent study demonstrated high reproducibility and reliability of the measurement of gingival recession using a virtual model produced by intraoral scanning [14]. Another study using an in vivo model also showed that digital scanning method in measuring KG is more accurate than conventional periodontal probing [15].

Therefore, anatomical information can be enhanced by digital technology. The present study aimed to investigate the gingival dimensions by digital scanning in periodontally healthy Korean individuals. Moreover, based on the collected data, ROC curves were estimated to determine the gingival dimension’s predictive value.

## 2. Materials and Methods

### 2.1. Study Design

The present study is a cross-sectional observational study. The study protocol was approved by the Dankook University Institutional Review Board at Cheonan Campus (IRB No. DKU-IRB 2019-06-005-001). The study was conducted between July and August 2019.

### 2.2. Study Population

The inclusion criteria were as follows: (1) volunteers from Dankook University Dental College, (2) age ≥20 years, (3) acceptable oral hygiene with full mouth plaque index <20% and full mouth bleeding index <20%, and (4) no missing teeth except for the third molar. The exclusion criteria were as follows: (1) history of orthodontic treatment, (2) malalignment of dentition (apparent deviation from the ideal arch line; score 1 and 2 according to the Malalignment Index system [16]), (3) current periodontal disease and history of periodontitis, (4) gingival recession (intraoral exposure of the cemento-enamel junction), (5) dental restorations potentially affecting the gingival margin, and (6) presence of dental implant(s).

### 2.3. Sampling

Convenience sampling was performed at Dankook University Dental College, assuming that dental students are more eager to oral hygiene practice than the general population. Consequently, fifty-one periodontally and systemically healthy volunteers (38 males and 13 females, mean age: 24.2 ± 2.1 years) were recruited.

### 2.4. Examination

All clinical examinations were performed by a single investigator (J.C.P.). The investigator has >10 years of experience as a board-certified periodontal specialist.

#### 2.4.1. Intraoral Scanning

**Figure 1:**
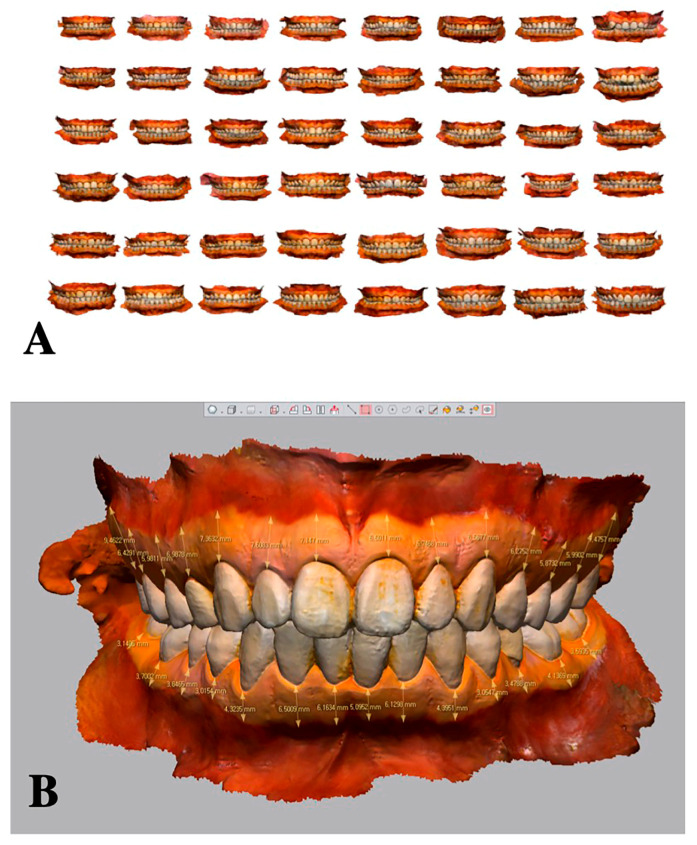
Intraoral scanning and measurement. (**A**) Intraoral scanning of the included participants, (**B**) measurement.

With a plastic dental cheek retractor (Hanil, Seoul, Korea), the accessibility and visibility for intraoral scanning were established. The gingiva was dried with an air syringe, and Lugol’s iodine solution (GEMST-S, 2.5%, Kormar Korea, Seoul, Korea) was applied to the buccal gingiva with cotton rolls; excessive solution was removed with dental suction, leading to a clear demarcation between the KG and alveolar mucosa (mucogingival junction [MGJ]). Then, an intraoral digital scanner (i500, iScan version 1.2.0.1; Medit, Seoul, Korea) was used for whole arch scanning. The extent of the scanning was up to the deepest portion of the vestibule. The same scanning procedure was used for all patients: The KG around the anterior region was first scanned. Then, the upper and the lower-left posterior regions were scanned with the examiner standing in the 12 o’clock position to the patient. Then, in the 7 o’clock position, the examiner scanned the upper and the lower-right posterior regions. The scanning procedure was conducted under the recommended conditions of level-1 filtering and a 21.0-mm scan depth (Figure 1A).

#### 2.4.2. Clinical Measurements

With a UNC-15 probe, the probing depth, plaque index [17], and gingival index [18] were measured. Plaque and gingival indices were recorded prior to intraoral scanning. After scanning, the probing depth was measured.

### 2.5. Analysis

After the digital scans were acquired, the scanned data in the polygon file format (PLY) were exported from the scanning program (Medit link, Medit). The dataset imported into the image analysis software (Geomagic Design X™, 3D Systems, Inc., Rock Hill, CA, USA) were visualized in 3D reconstructed images (Figure 1B). A single investigator (J.L.) who was trained in using the software calibrated it by measuring 10 random samples two times each in a span of one week under the supervision of a senior investigator (J.C.P.). All measurements were rounded to the nearest 0.01 mm. The calibration session yielded an intraclass correlation coefficient (ICC) of 0.90.

The following parameters were measured: (1) height of the KG: distance between the midfacial gingival margin and MGJ along the long axis of the tooth, (2) area of the KG: An imaginary line was drawn to divide each interdental papilla in half and the line was extended to the MGJ. Thus, the area of the KG for each tooth was established, and (3) height of AG: This was measured by subtracting the probing depth from the height of the KG.

### 2.6. Statistics

All measurements were recorded in an Excel spreadsheet (version 2009; Microsoft, Redmond, WA, USA). The data were analyzed using R studio statistical software (R 3.6.1). The Shapiro–Wilk test was applied for conformity of normal distribution of the data. Then, for each parameter of all teeth, the Mann–Whitney U test was used for statistical difference between male and female. Receiver operation characteristic (ROC) curves were estimated using a logistic regression model with cut-off values to differentiate between males and females on the basis of gingival dimensions. Subsequently, the area under the curve (AUC), sensitivity, specificity, and accuracy were calculated. The level of statistical significance was set at *p* < 0.05.

## 3. Results

Of the 51 volunteers (38 males and 13 females, mean age: 24.2 ± 2.1), three had missing teeth, so their data were excluded. Finally, 48 volunteers (38 males and 10 females, mean age: 24.3 ± 2.2) were included in the analysis. The data on tooth type and tooth number in both jaws are presented in Table 1, Table 2 and Table 3 and Table A1, Table A2 and Table A3, respectively.

### 3.1. Height of Keratinized Gingiva

**Figure 2:**
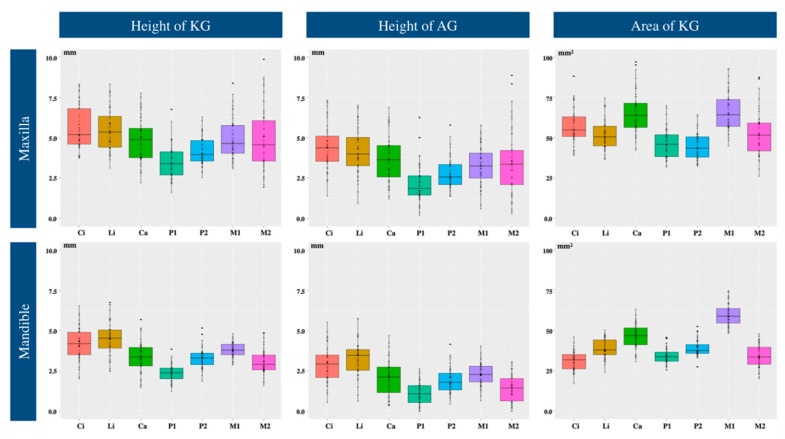
Whisker and box plots of the data sorted by tooth type in both jaws. KG: keratinized gingiva, AG: attached gingiva, Ci: central incisor, Li: lateral incisor, Ca: canine, P1: 1st premolar, P2: 2nd premolar, M1: 1st molar, M2: 2nd molar.

For each tooth position, the height of the KG was greater in the maxilla than in the mandible. The greatest and the least differences were observed in the second molar (4.56 mm (3.98, 5.23) in the maxilla vs. 2.90 mm (2.51, 3.10) in the mandible; data are presented as median value with 95% confidence interval) and in the second premolar (3.95 mm (3.62, 4.18) vs. 3.28 mm (3.15, 3.52)), respectively (Table 1, Figure 2).

In both jaws, similar trends of KG distribution were observed. The least value was noted in the first premolar. The values in this area were 3.39 mm (2.95, 3.75) in the maxilla and 2.37 mm (2.18, 2.55) in the mandible. The height of the KG in the central and lateral incisors was comparable in each jaw (5.17 mm (4.64, 5.51) and 5.33 mm (4.80, 5.87) in the maxilla, 4.18 mm (4.01, 4.37) and 4.52 mm (4.25, 4.80) in the mandible) and was higher than that in other areas of each jaw. In the maxilla, the height of the KG in the first (4.63 mm (4.04, 4.99)) and the second molars was similar (4.56 mm (3.98, 5.23)), but in the mandible, the value was greater in the first molar (3.78 mm (3.60, 3.86)) than in the second molar (2.90 mm (2.51, 3.10)). The height of the KG was not statistically significantly different between male and female volunteers (*p* > 0.05).

### 3.2. Height of Attached Gingiva

Same as the height of the KG, for each tooth position, the height of the AG was greater in the maxilla than in the mandible. In both jaws, the first premolar showed the lowest height (1.85 mm (1.38, 2.12) vs. 1.07 mm (0.79, 1.38)). In the maxilla, the median value of the AG height for all the teeth was over 2 mm except for the first premolar. However, in the mandible, the height in the first and second premolars (1.78 mm (1.45, 1.97)) and second molars (1.42 mm (1.22, 1.71)) was less than 2 mm (Table 2, Figure 2). Height of the AG was not statistically significantly different between male and female volunteers (*p* > 0.05).

### 3.3. Area of Keratinized Gingiva

In both jaws, the area of the KG was the largest in the first molar (64.14 mm^2^ (57.87, 69,47) in the maxilla, 58.82 mm^2^ (56.54, 60.98) in the mandible), followed by the canine (63.74 mm^2^ (57.33, 68.89), 46.85 mm^2^ (44.01. 48.91)). In the maxilla, the area in the premolars (45.78 mm^2^ (41.83, 49.58) and 43.42 mm^2^ (37.93, 46.50)) presented the smallest values compared to other areas (between 50.40 mm^2^ (47.00, 53.43) for the lateral incisor and 64.14 mm^2^ (57.87, 69,47) for the first molar). The values in the second molar (51.70 mm^2^ (45.85, 57.06)) and the lateral incisor were similar. In the mandible, the area in the central incisor was the smallest (31.71 mm^2^ (30.23, 35.03)). The difference in the area of the KG between the first and second molars was greater in the mandible (58.82 mm^2^ (56.64, 60.98) vs. 33.49 mm^2^ (30.40, 35.37)) than in the maxilla (64.14 mm^2^ (57.87, 69,47) vs. 51.70 mm^2^ (45.85, 57.06)) (Table 3, Figure 2). The area of the KG in the maxillary and mandibular canines and molars was statistically significantly different between male and female volunteers (*p* < 0.05).

### 3.4. Determination of Sex on a Basis of Gingival Dimensions

AUCs were calculated for the parameters of all teeth. Then, the parameters with AUC over 0.7 were selected as per the recommendations of Muller et al. (2005) [19]. In their study, 1.0–0.9 AUC was considered excellent, 0.9–0.8, good, 0.8–0.7, fair, 0.7–0.6, poor, and 0.6–0.5, fail. The selected parameters were area of the KG in the mandibular second molar (AUC = 0.74, cut-off value = 31.56 mm^2^), mandibular canine (AUC = 0.73, cut-off value = 40.47 mm^2^), maxillary canine (AUC = 0.72, cut-off value = 64.44 mm^2^), and mandibular first molar (AUC = 0.71, cut-off value = 53.1 mm^2^) (Table 4). For example, when the area of the KG on the mandibular second molar was over 31.56 mm^2^, the subject was likely to be male. The sensitivity, specificity, and accuracy are presented in Table 4.

## 4. Discussion

The gingiva has a convex curvilinear shape. In the past, this curvilinear outline could not be recorded the way it is; the measurements from a straight line connecting two landmarks were used to determine a representative value. Until now, no quantitative information regarding curvilinear gingival line was reported. Considering this, the present study investigated the gingival dimensions of periodontally healthy young adults with the use of an intraoral scanner. The current investigation can help to enhance anatomic information with high accuracy.

In general, the distribution pattern of the gingival height in the present study was in conformity to previous studies. A comparison of the maxilla and mandible revealed that the height of the KG and AG were higher in maxillary teeth than in the mandibular teeth in the same position. The height of the KG and AG in both jaws showed similar fluctuation trends by tooth type (See Figure 2). Regarding the maxillary central and maxillary lateral incisors, little heterogeneity was found in the gingival distribution pattern between different studies (including the present study). In the present study, the gingival heights in the maxillary central incisor (5.17 mm for KG, 4.35 mm for AG; median value) showed different tendency from those in the maxillary lateral incisor (5.33 mm for KG, 3.98 mm for AG). This tendency (depending on KG or AG or both) is in line with the findings of the study by Adesola et al. [20] but not of Bower [4], Ainamo and Loë [1] and Shirmohammadi, Faramarzi, Lafzi [21] (see the values of these studies in Table A4).

In both jaws, the lowest KG height was reported in the first premolar (3.39 mm in the maxilla, 2.37 mm in the mandible), which resulted in the low height of the AG in this position. Especially, the height of AG in the mandibular second premolar was approximately 1 mm, which is a striking finding compared to that of other studies performed in different countries (1.8–2.42 mm) (See Table A4) [1,2,4,20,21,22]. Frenular attachment adjacent to the premolar appears to affect the height of the AG.

Interestingly, the height of AG in the mandibular second molar was also small (approximately 1.0 mm) in the present study. Anatomically, the external oblique ridge starts from third molar area, leading to the extension of the buccal mucosa to the mandibular second molar. Owing to this, the height of the KG in the mandibular second molar tends to be smaller than that in the mandibular first molar and maxillary second molar, and the height of the AG changes concomitantly. It is also noteworthy that the height of the AG in the mandibular second molar of this study was lower compared to other studies [1,2,4,20,21,22]. This difference might be attributable to the difference in jaw growth and mucosal attachment.

The area of the KG in each tooth position was firstly reported in the present study. The different mesio-distal widths of each tooth considerably affected the area of the KG. For example, the canine and the first molar had larger KG area than the other teeth. The area of the KG may influence the mechanical stability of the soft tissue, such as susceptibility to the recession. However, epidemiologic studies demonstrated heterogeneous findings [23,24,25].

The heights of the KG and AG in the Korean population are smaller than the values reported in other studies [1,2,4,20,21,22]. Previously, the KG and/or AG were measured in the US [4], Denmark [1], Iran [21], India [22], Nigeria [20]. Especially, the height of the AG in the Iranian population was notably greater than that in other populations [21]. This indicates ethnic and racial differences in the dimensions of the KG and AG, which was also discussed in other studies [20,21]. Other reasons behind this difference may be as follows: (1) The age groups targeted were different across the studies. Previously, an increase in the AG was found with age [26], owing to the constant position of the MGJ and attrition-related tooth eruption. (2) Digital measurements provide different levels of accuracy as opposed to conventional measurements. A recent study comparing histological and digital/clinical measurements in a pig jaw model showed that the digital technique had superior accuracy and reliability in measuring the KG than the periodontal probe [15]. (3) Periodontal situation, a history of orthodontic treatment, tooth malposition was inconsistent or not reported.

A systematic review investigating the correlation between periodontal phenotype and gingival dimensions showed that the mean KG height ranged between 2.75 and 5.44 mm in the thin phenotype and between 5.09 mm and 6.65 mm in the thick phenotype [27]. According to this, most of the present study subjects might fall under the thin phenotype, which may be in line with recent evidence that Asian population has a thin gingival phenotype compared to white population [9]. However, this speculation should be back up by further data from other measurements, such as cone-beam computed tomography, ultrasonography, or endodontic instruments [28].

In the present study, we tried to differentiate between male and female volunteers using the dimensional data. AUC > 0.7 was reported for the area of the KG in the mandibular second molar, mandibular canine, maxillary canine, and mandibular first molar. Among those, the mandibular second molar showed the highest sensitivity, and the mandibular canine and first molar showed the highest specificity. The dataset was further used for machine learning tools, such as random forest and Xgboost, despite the small sample size. However, some of the data from female volunteers were spent in the training set, which led to less data in the test set. To draw more reliable results from machine learning, a larger dataset should be collected from both male and female.

There are some limitations and considerations in the present study. First, gingival phenotype was not recorded, as mentioned above. The addition of such data would provide more comprehensive information about the gingival tissues. Second, there was a discrepancy between the numbers of male and female subjects, which might affect statistical results. Third, the pattern of gingival dimension in the present study was not different from that in the previous studies, indicating traditional measurement is still valid when high inter/ intra-examiner reproducibility and reliability are obtained.

Several things should be covered in future studies, such as comparing between measurements using a periodontal probe and using digital scanning, gingival dimensions with/without periodontal diseases, and gingival dimension change over time using superimposition. Especially, the data from digital scanning technology can be utilized to visualize the change for the area of the investigators’ interest and measure profilometric change in great detail, which was not feasible using traditional methods.

## 5. Conclusions

In conclusion, the gingival dimensions recorded using an intraoral scanner showed a similar distribution pattern as that in previous studies. However, the dimensions appear to be influenced by race and/or ethnicity, especially in the mandibular canine and second molar. Some of the gingival dimensions may be used for differentiating between male and female.

## Figures and Tables

**Table 1:** Height of the keratinized gingiva (in mm).

	Jaw	Maxilla							Mandible						
	Tooth type	Ci	Li	Ca	P1	P2	M1	M2	Ci	Li	Ca	P1	P2	M1	M2
Total (*n* = 48)	Mean	5.60	5.49	4.87	3.50	4.14	4.91	4.88	4.17	4.50	3.35	2.36	3.27	3.79	3.04
	SD	1.36	1.29	1.36	1.05	0.86	1.25	1.93	0.99	1.04	0.93	0.54	0.61	0.45	0.73
	Median	5.17	5.33	4.88	3.39	3.95	4.63	4.56	4.18	4.52	3.34	2.37	3.28	3.78	2.90
	Inter-quartile range	2.22	2.01	1.87	1.45	1.31	1.74	2.57	1.41	1.16	1.16	0.70	0.72	0.68	0.94
	95% CI	4.64, 5.51	4.80, 5.87	4.44, 5.75	2.95, 3.75	3.62, 4.18	4.04, 4.99	3.98, 5.23	4.01, 4.37	4.25, 4.80	3.00, 3.57	2.18, 2.55	3.15, 3.52	3.60, 3.86	2.51, 3.10
Male (*n* = 38)	Mean	5.65	5.59	4.96	3.51	4.12	4.85	4.97	4.31	4.64	3.45	2.29	3.28	3.84	3.10
	SD	1.27	1.33	1.44	1.07	0.91	1.27	2.02	0.91	1.01	0.83	0.50	0.56	0.38	0.77
	Median	5.23	5.40	5.00	3.43	3.83	4.47	4.56	4.24	4.52	3.42	2.35	3.28	3.80	3.08
	Inter-quartile range	2.22	1.95	2.50	1.45	1.41	1.79	2.68	1.30	1.03	1.08	0.80	0.61	0.54	0.97
	95% CI	4.65, 5.63	4.90, 6.02	4.55, 5.87	2.96, 3.80	3.33, 4.00	4.01, 4.73	3.91, 5.10	4.00, 4.46	4.17, 4.77	3.00, 3.61	2.13, 2.54	3.05, 3.50	3.60, 3.87	2.77, 3.42
Female (*n* = 10)	Mean	5.40	5.09	4.53	3.43	4.23	5.16	4.56	3.66	4.00	2.98	2.61	3.24	3.58	2.83
	SD	1.71	1.13	0.96	1.03	0.63	1.22	1.58	1.13	1.08	1.22	0.65	0.79	0.65	0.53
	Median	4.98	5.07	4.14	3.06	3.99	5.23	4.37	3.36	4.21	2.76	2.41	3.17	3.40	2.66
	Inter-quartile range	2.53	1.93	1.76	1.85	1.08	1.68	2.18	1.63	1.62	1.35	1.17	0.64	1.01	0.26
	95% CI	2.85, 6.07	4.15, 6.17	2.84, 4.60	1.57, 3.54	3.18, 4.26	4.67, 6.39	2.95, 5.58	2.07, 4.01	3.66, 5.41	1.86, 3.52	1.57, 2.75	3.01, 3.65	2.72, 3.73	2.35, 2.81
*p*-value (male vs. female)		0.5044	0.3091	0.4279	0.9597	0.4885	0.4730	0.6957	0.0932	0.1581	0.1030	0.3334	0.5205	0.0932	0.1692

Ci: central incisor, Li: lateral incisor, Ca: canine, P1: 1st premolar, P2: 2nd premolar, M1: 1st molar, M2: 2nd molar, CI: confidence interval.

**Table 2:** Height of the attached gingiva (in mm).

	Jaw	Maxilla							Mandible						
	Tooth type	Ci	Li	Ca	P1	P2	M1	M2	Ci	Li	Ca	P1	P2	M1	M2
Total (*n* = 48)	Mean	4.38	4.19	3.69	2.14	2.79	3.27	3.43	2.88	3.27	2.03	1.08	1.90	2.29	1.40
	SD	1.42	1.44	1.48	1.16	0.98	1.22	2.00	1.11	1.07	1.10	0.72	0.78	0.70	0.84
	Median	4.35	3.98	3.62	1.85	2.56	3.25	3.35	2.91	3.45	2.11	1.07	1.78	2.27	1.42
	Inter-quartile range	1.63	1.87	2.04	1.21	1.26	1.70	2.13	1.41	1.32	1.62	1.06	1.07	1.00	1.42
	95% CI	4.08, 4.95	3.31, 4.29	3.01, 4.26	1.38, 2.12	2.25, 2.75	2.79, 3.67	3.04, 4.14	2.62, 3.35	3.23, 3.92	1.80, 2.55	0.79, 1.38	1.45, 1.97	1.99, 2.43	1.22, 1.71
Male (*n* = 38)	Mean	4.44	4.31	3.80	2.16	2.79	3.28	3.58	2.99	3.41	2.10	1.08	1.92	2.40	1.49
	SD	1.26	1.37	1.56	1.20	1.04	1.27	2.10	1.07	0.98	1.04	0.71	0.75	0.64	0.84
	Median	4.38	3.98	3.71	1.85	2.56	3.21	3.37	2.99	3.47	2.20	1.01	1.85	2.28	1.58
	Inter-quartile range	1.21	1.61	2.40	1.24	1.26	1.69	2.01	1.31	1.22	1.35	1.08	0.93	0.95	1.15
	95% CI	4.06, 5.00	3.11, 4.25	2.92, 4.52	1.19, 2.13	2.14, 2.85	2.73, 3.63	2.99, 3.77	2.65, 3.47	3.18, 3.92	1.96, 2.55	0.60, 1.37	1.56, 2.09	1.84, 2.35	1.36, 2.03
Female (*n* = 10)	Mean	4.15	3.74	3.28	2.05	2.78	3.21	2.86	2.46	2.75	1.73	1.08	1.79	1.89	1.07
	SD	1.99	1.64	1.06	1.08	0.73	1.03	1.53	1.20	1.27	1.30	0.81	0.91	0.81	0.75
	Median	3.73	3.63	3.02	1.99	2.61	3.52	2.37	2.11	2.71	1.49	1.22	1.48	1.87	1.16
	Inter-quartile range	2.69	2.79	1.54	1.09	1.37	1.76	2.83	1.77	1.76	1.64	0.70	1.15	0.99	0.96
	95% CI	2.04, 4.85	1.88, 4.66	1.83, 3.60	1.10, 2.72	1.76, 3.13	3.07, 4.84	0.50, 3.33	0.88, 2.65	1.59, 3.66	0.68, 2.32	0.81, 1.90	0.48, 1.86	1.39, 2.47	0.83, 1.85
*p*-value (male vs. female)		0.4730	0.3589	0.3721	0.9698	0.8105	0.9698	0.4426	0.1442	0.1581	0.2751	0.9095	0.3994	0.0886	0.1476

Ci: central incisor, Li: lateral incisor, Ca: canine, P1: 1st premolar, P2: 2nd premolar, M1: 1st molar, M2: 2nd molar, CI: confidence interval.

**Table 3:** Area of the keratinized gingiva (in mm^2^).

	Jaw	Maxilla							Mandible						
	Tooth type	Ci	Li	Ca	P1	P2	M1	M2	Ci	Li	Ca	P1	P2	M1	M2
Total (*n* = 48)	Mean	57.19	51.77	65.33	46.44	44.57	65.95	53.51	31.09	38.83	46.48	34.12	39.07	59.74	34.16
	SD	10.78	9.20	13.06	9.01	8.14	11.77	15.82	5.92	6.03	7.47	4.94	4.85	7.12	6.97
	Median	54.76	50.40	63.74	45.78	43.42	64.14	51.70	31.71	37.85	46.85	33.61	37.50	58.82	33.49
	Inter-quartile range	12.39	12.54	15.40	13.69	12.75	16.86	17.62	9.15	9.47	10.88	5.89	5.95	9.35	11.00
	95% CI	49.43, 56.93	47.00, 53.43	57.33, 68.89	41.83, 49.58	37.93, 46.50	57.87, 69.47	45.85, 57.06	30.23, 35.03	35.21, 38.72	44.01, 48.91	32.44, 35.34	34.96, 38.25	56.54, 60.98	30.40, 35.37
Male (*n* = 38)	Mean	58.30	52.52	67.37	47.07	45.00	66.30	54.93	31.79	39.40	47.68	33.69	39.00	60.63	35.25
	SD	9.99	9.56	13.28	9.36	8.20	12.57	17.03	5.77	5.96	7.11	4.76	4.45	6.45	6.85
	Median	55.24	50.89	66.91	47.68	43.42	63.08	52.35	32.01	38.12	48.49	33.02	38.17	60.11	34.49
	Inter-quartile range	11.89	12.61	18.69	13.91	11.70	18.48	19.25	8.90	10.51	9.53	5.64	6.62	7.69	10.25
	95% CI	49.28, 57.86	47.03, 54.18	62.85, 74.17	44.52, 52.28	38.66, 46.19	55.61, 67.47	45.96, 58.47	30.24, 34.93	34.64, 38.88	46.48, 51.70	31.13, 34.69	36.03, 39.59	57.62, 62.10	30.48, 36.05
Female (*n* = 10)	Mean	53.00	48.94	57.54	44.04	42.91	64.61	48.11	28.44	36.69	41.91	35.73	39.31	56.34	30.01
	SD	13.10	7.46	8.97	7.48	8.12	8.47	8.64	6.01	6.12	7.41	5.52	6.43	8.83	6.04
	Median	53.37	48.34	57.88	41.98	43.49	64.50	47.84	25.16	35.33	39.91	34.17	36.74	52.65	27.61
	Inter-quartile range	19.69	10.88	13.42	6.12	13.63	13.77	17.19	10.55	11.17	11.63	6.14	4.62	8.75	6.46
	95% CI	42.61, 66.24	41.10, 54.03	51.46, 65.10	34.53, 44.87	36.61, 51.53	57.20, 71.89	39.20, 56.39	16.06, 26.81	27.43, 38.59	33.15, 44.78	28.63, 36.71	30.07, 38.17	41.05, 55.42	21.73, 29.31
*p*-value (male vs. female)		0.2151	0.3211	0.0371 *	0.3460	0.5370	0.8696	0.2438	0.0886	0.1510	0.0331 *	0.4279	0.4885	0.0466 *	0.0246 *

Ci: central incisor, Li: lateral incisor, Ca: canine, P1: 1st premolar, P2: 2nd premolar, M1: 1st molar, M2: 2nd molar, CI: confidence interval, * significantly different between male and female.

**Table 4:** Area under the receiver operation characteristics curve, sensitivity, specificity and accuracy of the selected teeth.

Parameter	AUC	Cut-off Value	Sensitivity	Specificity	Accuracy
The area of KG on the mandibular second molar	0.74	31.56 mm^2^	0.7	0.74	0.73
The area of KG on the mandibular canine	0.73	40.47 mm^2^	0.6	0.89	0.83
The area of KG on the maxillary canine	0.72	64.44 mm^2^	0.8	0.55	0.6
The area of KG on the mandibular first molar	0.71	53.10 mm^2^	0.6	0.89	0.83

KG: keratinized gingiva.

## Data Availability

The data presented in this study are available on request from the corresponding author. The data are not publicly available due to patients’ privacy.

## Appendix A

**Table A1:** Height of the keratinized gingiva (in mm).

	Tooth Number	17	16	15	14	13	12	11	21	22	23	24	25	26	27	47	46	45	44	43	42	41	31	32	33	34	35	36	37
Total (*n* = 48)	Average	4.87	4.99	4.09	3.43	4.73	5.52	5.66	5.54	5.45	5.01	3.56	4.19	4.84	4.89	3.03	3.81	3.31	2.45	3.38	4.49	4.17	4.18	4.52	3.33	2.27	3.23	3.77	3.06
	SD	2.08	1.37	1.00	1.19	1.47	1.41	1.45	1.37	1.34	1.55	1.22	0.92	1.33	2.21	0.87	0.61	0.70	0.61	1.12	1.16	1.07	1.07	1.07	0.85	0.61	0.68	0.45	0.74
	Median	4.33	4.81	3.85	3.16	4.44	5.10	5.22	5.20	5.36	5.12	3.24	4.16	4.58	4.56	2.94	3.85	3.27	2.55	3.26	4.47	4.14	4.13	4.60	3.36	2.24	3.25	3.78	3.05
	Inter-quartile range	3.01	1.96	0.94	1.53	2.22	2.00	2.80	1.95	2.19	2.39	1.68	1.16	1.57	2.51	0.99	0.89	0.80	0.93	1.21	1.30	1.76	1.31	1.25	1.15	0.70	0.75	0.52	1.02
	95% CI	3.66, 5.04	4.29, 5.36	3.56, 4.10	2.57, 3.50	3.96, 4.88	4.25, 5.36	4.37, 5.68	4.83, 5.63	4.72, 5.99	4.49, 6.11	2.38, 3.59	3.82, 4.52	4.05, 4.83	3.98, 5.33	2.58, 3.11	3.58, 4.15	3.11, 3.43	2.33, 2.90	2.69, 3.47	4.23, 4.81	3.75, 4.63	3.84, 4.45	4.31, 4.95	3.14, 3.51	2.03, 2.41	3.14, 3.53	3.67, 3.90	2.74, 3.40
Male (*n* = 38)	Average	4.93	4.93	4.07	3.43	4.86	5.60	5.73	5.58	5.58	5.06	3.59	4.16	4.78	5.01	3.05	3.85	3.29	2.38	3.54	4.59	4.31	4.31	4.69	3.36	2.21	3.26	3.84	3.15
	SD	2.23	1.42	1.06	1.21	1.56	1.44	1.38	1.27	1.38	1.65	1.28	0.97	1.31	2.31	0.96	0.52	0.70	0.58	0.95	1.14	1.02	1.01	1.02	0.82	0.57	0.60	0.38	0.72
	Median	4.28	4.62	3.81	3.16	4.44	5.10	5.35	5.16	5.43	5.12	3.54	4.08	4.51	4.56	2.95	3.90	3.29	2.51	3.61	4.53	4.33	4.17	4.68	3.40	2.24	3.28	3.84	3.20
	Inter-quartile range	3.04	1.99	1.02	1.47	2.92	1.92	2.73	2.01	2.25	2.34	1.65	1.19	1.51	3.04	1.05	0.71	0.81	0.96	1.13	1.23	1.74	1.26	1.19	1.22	0.77	0.60	0.49	0.98
	95% CI	3.06, 4.94	3.91, 5.22	3.49, 4.01	2.57, 3.61	3.37, 4.89	4.10, 5.42	4.52, 5.84	4.48, 5.51	4.64, 5.95	4.34, 6.12	2.90, 4.14	3.64, 4.43	3.92, 4.71	3.85, 5.35	2.53, 3.16	3.67, 4.08	3.07, 3.54	2.30, 2.91	3.34, 4.09	4.31, 4.88	3.82, 4.90	3.90, 4.52	4.27, 4.96	3.13, 3.78	2.05, 2.47	3.18, 3.56	3.74, 3.98	2.98, 3.53
Female (*n* = 10)	Average	4.67	5.22	4.16	3.44	4.24	5.23	5.42	5.39	4.95	4.82	3.42	4.30	5.09	4.45	2.94	3.64	3.35	2.71	2.76	4.13	3.66	3.65	3.87	3.19	2.51	3.13	3.52	2.72
	SD	1.49	1.18	0.77	1.17	1.02	1.34	1.75	1.76	1.09	1.16	1.00	0.74	1.42	1.85	0.39	0.87	0.74	0.67	1.51	1.21	1.16	1.18	1.04	1.02	0.74	0.95	0.61	0.77
	Median	4.58	5.29	4.02	3.21	4.42	5.20	4.65	5.30	4.45	4.66	2.99	4.22	5.05	4.50	2.91	3.33	3.27	2.75	2.63	4.23	3.69	3.24	3.97	3.24	2.27	2.92	3.59	2.52
	Inter-quartile range	1.65	1.37	0.72	1.41	0.88	2.04	3.34	2.63	2.01	2.05	1.99	1.04	1.66	2.43	0.43	1.06	0.65	1.06	1.82	1.73	1.46	1.96	1.54	1.07	1.24	0.81	1.14	0.53
	95% CI	3.33, 5.77	4.45, 6.13	3.22, 4.51	1.86, 3.83	3.80, 5.60	4.35, 6.39	2.05, 5.39	3.96, 6.76	2.76, 4.77	3.56, 5.69	1.27, 3.29	3.59, 4.70	4.23, 6.16	3.24, 6.00	2.64, 3.12	2.45, 3.65	2.96, 3.72	2.16, 3.36	1.83, 3.84	3.45, 5.17	2.71, 4.86	1.83, 3.79	3.34, 4.99	2.58, 4.27	1.39, 2.62	1.99, 3.39	3.18, 4.32	2.04, 2.75
*p*-value (male vs. female)		0.9698	0.4135	0.7524	0.8498	0.3460	0.5044	0.3460	0.6052	0.1654	0.6864	0.7910	0.7144	0.5206	0.6772	0.7428	0.1849	0.9698	0.2017	0.0597	0.3856	0.1313	0.1252	0.0614	0.4279	0.3589	0.3091	0.1082	0.0759

FDI numbering system is used for indicating each tooth.

**Table A2:** Height of the attached gingiva (in mm).

	Tooth Number	17	16	15	14	13	12	11	21	22	23	24	25	26	27	47	46	45	44	43	42	41	31	32	33	34	35	36	37
Total (*n* = 48)	Average	3.41	3.36	2.69	2.07	3.63	4.17	4.41	4.35	4.22	3.76	2.21	2.88	3.18	3.44	1.41	2.33	1.89	1.09	2.09	3.24	2.84	2.93	3.31	1.96	1.08	1.90	2.25	1.39
	SD	2.27	1.48	1.14	1.34	1.68	1.69	1.55	1.42	1.47	1.66	1.29	1.07	1.31	2.26	0.96	0.88	0.90	0.77	1.33	1.15	1.05	1.35	1.18	1.05	0.82	0.95	0.76	0.93
	Median	2.83	3.56	2.56	1.93	3.31	3.98	4.30	4.16	4.21	3.74	2.09	2.62	3.13	3.22	1.45	2.36	1.80	1.00	2.07	3.18	2.82	2.77	3.47	2.20	1.08	1.88	2.29	1.55
	Inter-quartile range	2.46	2.06	1.37	1.47	2.61	2.23	2.00	1.49	1.98	2.72	1.72	1.17	1.30	2.95	1.17	1.39	1.18	1.35	1.63	1.43	1.64	1.49	1.45	1.41	1.02	1.22	0.86	1.64
	95% CI	2.05, 3.20	3.34, 4.19	2.12, 2.90	1.67, 2.52	2.72, 3.78	3.33, 4.55	3.58, 4.88	3.75, 4.66	3.57, 4.76	3.10, 4.36	1.78, 2.52	2.29, 2.84	2.85, 3.42	2.82, 3.99	1.20, 1.91	1.86, 2.62	1.42, 2.09	0.47, 1.30	1.85, 2.33	2.77, 3.56	2.33, 3.24	2.37, 3.10	3.20, 4.03	2.01, 2.73	0.91, 1.41	1.42, 2.38	2.01, 2.54	1.23, 2.05
Male (*n* = 38)	Average	3.51	3.35	2.70	2.09	3.76	4.26	4.49	4.40	4.37	3.85	2.23	2.87	3.22	3.65	1.44	2.43	1.95	1.10	2.25	3.35	2.96	3.03	3.47	1.96	1.07	1.90	2.36	1.54
	SD	2.37	1.53	1.20	1.40	1.79	1.69	1.37	1.29	1.36	1.73	1.35	1.13	1.33	2.38	1.00	0.81	0.88	0.72	1.23	1.03	1.01	1.34	1.15	1.04	0.83	0.92	0.72	0.90
	Median	2.93	3.43	2.60	1.84	3.31	3.98	4.30	4.16	4.36	3.89	2.09	2.62	3.23	3.45	1.45	2.49	1.85	1.00	2.20	3.27	2.89	2.91	3.58	2.21	1.01	1.95	2.32	1.75
	Inter-quartile range	3.13	2.40	1.51	1.61	3.47	2.08	1.83	1.26	1.86	2.76	1.78	1.39	1.18	3.34	1.23	1.38	1.20	1.34	1.51	1.36	1.49	1.31	1.32	1.04	1.07	1.20	0.92	1.38
	95% CI	2.14, 3.34	3.06, 4.07	2.11, 3.11	1.44, 2.45	2.13, 3.65	3.13, 4.49	3.54, 4.73	3.75, 4.60	3.86, 4.91	2.92, 4.79	1.52, 2.53	1.96, 2.85	2.98, 3.69	3.06, 4.39	1.18, 1.94	2.04, 2.79	1.41, 2.16	0.44, 1.32	1.61, 2.49	2.94, 3.71	2.37, 3.38	2.55, 3.32	3.23, 4.10	1.99, 2.70	0.74, 1.39	1.54, 2.51	1.97, 2.50	1.53, 2.14
Female (*n* = 10)	Average	3.07	3.42	2.66	1.98	3.14	3.83	4.12	4.19	3.65	3.42	2.12	2.90	2.99	2.65	1.31	1.94	1.65	1.03	1.47	2.83	2.36	2.55	2.67	1.99	1.12	1.93	1.84	0.83
	SD	1.89	1.37	0.94	1.15	1.08	1.74	2.18	1.89	1.81	1.37	1.07	0.87	1.27	1.57	0.84	1.08	0.97	0.97	1.57	1.51	1.13	1.40	1.13	1.13	0.83	1.08	0.82	0.85
	Median	2.43	3.61	2.52	2.02	3.04	3.70	3.88	3.80	3.39	3.51	1.93	2.61	3.10	2.60	1.28	1.74	1.63	1.14	1.13	2.91	2.19	2.24	2.69	1.78	1.14	1.56	1.92	0.63
	Inter-quartile range	2.18	1.39	1.32	1.23	1.41	2.28	3.58	2.70	2.08	1.56	1.57	0.72	1.42	1.98	0.94	1.22	1.52	1.44	1.86	2.43	1.52	2.08	2.23	1.37	0.65	1.21	1.00	1.45
	95% CI	0.86, 3.04	2.89, 4.28	1.83, 3.15	1.35, 2.81	2.00, 3.89	1.79, 4.99	1.52, 5.16	1.96, 5.02	1.64, 4.55	2.51, 4.64	0.84, 2.69	1.51, 2.92	2.22, 4.17	1.21, 3.76	0.73, 1.89	0.91, 2.36	0.96, 2.49	0.47, 1.94	0.12, 2.01	1.79, 4.22	1.21, 2.93	0.77, 2.96	1.79, 4.03	0.71, 2.51	0.82, 1.72	0.26, 2.02	1.26, 2.44	0.18, 1.26
*p*-value (male vs. female)		0.6052	0.7620	0.8498	0.8896	0.3856	0.5537	0.5044	0.4730	0.2339	0.4885	0.9497	0.8696	0.7620	0.2643	0.8497	0.0842	0.4730	0.9095	0.0799	0.4135	0.1252	0.2151	0.0720	0.8696	0.9094	0.8896	0.0956	0.0339 *

FDI numbering system is used for indicating each tooth. * significantly different between males and females.

**Table A3:** Area of the keratinized gingiva (in mm^2^).

	Tooth Number	17	16	15	14	13	12	11	21	22	23	24	25	26	27	47	46	45	44	43	42	41	31	32	33	34	35	36	37
Total (*n* = 48)	Average	51.89	65.84	44.89	45.16	65.94	52.65	57.31	57.08	50.89	64.71	47.72	44.24	66.06	55.14	35.33	59.72	39.07	34.33	47.79	39.53	30.10	32.09	38.14	45.16	33.91	39.07	59.76	32.98
	SD	18.85	13.44	9.27	9.29	14.28	10.56	11.58	10.66	9.46	14.15	10.72	8.68	12.08	18.27	9.20	8.64	6.10	4.88	8.38	6.96	5.85	6.48	6.00	7.61	6.34	5.05	6.98	6.50
	Median	47.63	64.51	45.45	42.67	61.96	52.97	55.17	55.29	49.89	62.93	46.91	44.08	64.60	53.38	37.55	58.88	37.78	33.34	47.49	38.61	31.20	31.58	37.09	45.03	32.94	38.55	59.80	32.83
	Inter-quartile range	18.41	14.89	11.56	14.97	19.57	13.61	14.41	13.27	12.49	18.48	10.68	12.68	18.34	19.72	13.09	10.58	7.99	6.27	12.66	10.27	8.67	9.72	8.79	10.41	7.18	5.56	10.59	8.50
	95% CI	41.43, 50.64	61.80, 69.06	43.75, 50.40	36.45, 44.34	54.77, 67.13	50.97, 56.95	50.23, 58.44	50.37, 57.14	47.08, 54.23	56.51, 67.10	44.63, 50.50	40.26, 47.83	59.23, 68.45	48.08, 59.33	35.40, 44.02	57.06, 61.37	34.19, 39.66	31.01, 35.05	44.69, 50.05	35.08, 41.20	29.62, 34.89	28.96, 33.70	33.54, 38.76	41.11, 47.68	31.77, 34.68	37.19, 39.41	57.84, 62.51	30.98, 34.55
Male (*n* = 38)	Average	53.01	65.83	45.70	45.85	68.12	53.23	58.42	58.17	51.80	66.63	48.30	44.30	66.77	56.85	36.54	60.71	39.41	33.84	49.16	40.00	30.83	32.75	38.79	46.19	33.55	38.60	60.56	33.96
	SD	20.49	14.43	9.30	9.66	14.67	11.16	10.81	9.98	9.58	14.54	11.29	8.48	12.64	19.64	9.48	8.16	5.71	4.55	7.94	6.97	5.60	6.36	6.01	7.21	6.48	4.79	6.52	6.07
	Median	48.29	64.51	45.53	44.87	67.93	53.05	55.17	57.02	50.93	63.88	48.06	43.41	64.60	55.40	39.00	59.57	38.95	33.34	49.24	39.78	31.65	32.13	37.63	47.80	32.94	38.43	60.23	33.54
	Inter-quartile range	18.83	18.78	12.18	15.11	24.22	13.88	13.52	10.50	11.30	21.17	12.79	10.39	20.46	22.54	12.79	7.59	7.58	5.33	13.32	10.21	7.06	7.63	7.28	9.69	8.97	5.66	9.17	9.13
	95% CI	41.66, 51.86	62.02, 70.60	43.06, 50.60	39.58, 48.79	63.32, 79.01	49.20, 57.23	49.47, 58.40	52.27, 60.26	47.58, 53.32	55.66, 67.38	46.55, 52.36	38.61, 46.49	57.75, 68.60	49.27, 64.56	37.85, 44.45	57.59, 61.34	35.65, 41.34	31.12, 35.05	46.51, 52.90	35.68, 43.55	30.47, 34.97	29.10, 34.26	34.51, 39.15	46.10, 52.60	31.57, 35.12	36.96, 39.51	58.26, 63.32	30.68, 35.34
Female (*n* = 10)	Average	47.61	65.87	41.81	42.55	57.65	50.46	53.08	52.93	47.41	57.43	45.53	44.02	63.34	48.62	30.76	55.95	37.76	36.18	42.58	37.72	27.28	29.60	35.66	41.25	35.28	40.86	56.72	29.26
	SD	10.16	9.28	8.96	7.62	9.20	7.94	13.95	12.66	8.59	10.10	8.32	9.86	9.78	9.81	6.60	9.78	7.61	5.85	8.34	6.95	6.21	6.66	5.55	8.20	5.92	5.86	8.16	7.05
	Median	46.97	65.47	41.15	41.22	58.58	50.94	53.25	51.70	43.75	57.19	44.43	45.02	64.30	48.51	29.74	52.04	35.51	34.87	40.47	37.22	23.41	26.96	33.15	39.73	33.02	39.00	53.27	29.40
	Inter-quartile range	16.87	13.11	13.47	7.40	9.09	9.32	21.95	17.51	10.83	15.86	11.20	17.49	13.40	8.89	10.22	6.83	4.43	7.67	10.39	10.31	11.01	9.63	10.98	12.59	6.53	4.16	10.79	9.48
	95% CI	35.17, 53.57	60.28, 73.39	34.39, 48.29	34.51, 45.54	52.03, 63.73	44.68, 57.31	45.52, 67.47	40.71, 61.65	32.15, 46.12	50.17, 66.95	39.89, 51.17	36.93, 55.63	58.31, 72.80	42.10, 54.81	23.38, 34.39	40.45, 55.46	26.66, 37.84	30.81, 38.53	33.55, 44.46	31.36, 41.88	12.58, 24.04	19.22, 29.17	23.95, 34.99	30.06, 45.58	27.32, 35.52	34.37, 40.68	43.35, 55.54	26.65, 36.22
*p*-value (male vs. female)		0.6229	0.5878	0.3091	0.3721	0.0932	0.6229	0.2975	0.1252	0.1252	0.0886	0.3994	0.9899	0.5878	0.2643	0.0466 *	0.0331 *	0.2339	0.3091	0.0294 *	0.2975	0.1313	0.1137	0.1193	0.0842	0.5370	0.4279	0.1137	0.0351 *

FDI numbering system is used for indicating each tooth. * significantly different between males and females.

**Table A4:** Comparison of the gingival dimensions in previous studies.

			Maxilla							Mandible						
Study	Country	Value	Ci	Li	Ca	P1	P2	M1	M2	Ci	Li	Ca	P1	P2	M1	M2
Bowers (1963) [4] (AG)	Denmark	Mean (right/left quadrant)	4.7 4.6	5.2 5.1	4.0 3.8	3.6 3.5	4.1 4.2	4.1 4.2	3.9 4.1	2.9 2.7	3.5 3.3	2.0 2.1	2.0 2.0	2.5 2.5	2.8 2.7	2.3 2.2
Ainamo and Loë (1966) [1] (AG)	U.S.	In case of GM on enamel Mean (range)	3.5 (0–6)	4.5 (2–7)	2.7 (0–6)	1.9 (0–6)	2.7 (1–5)	3.0 (1–6)	2.9 (0–5)	3.3 (1–6)	3.9 (1–6)	2.6 (1–5)	1.8 (0–4)	2.1 (1–4)	2.4 (0–4)	1.9 (0–3)
		In case of GM on cementum Mean (range)	4.3 (0–6)	4.3 (0–6)	2.9 (0–6)	2.2 (1–4)	2.4 (1–5)	2.1 (0–3)	2.3 (1–4)	2.9 (1–5)	3.6 (2–6)	2.4 (0–4)	1.8 (0–6)	1.7 (1–4)	2.0 (1–4)	2.0 (1–3)
Shirmohammadi, Faramarzi, Lafzi (2008) [21] (KG/AG)	Iran	KG Mean ± SD (right/left quadrant)	6.24 ± 1.60 6.21 ± 1.75	6.93 ± 1.80 6.50 ± 1.64	5.99 ± 1.97 6.03 ± 1.90	4.77 ± 1.48 5.01 ± 1.71	5.04 ± 1.34 4.88 ± 1.42	5.70 ± 1.49 5.33 ± 1.38	5.67 ± 1.54 5.45 ± 1.49	4.33 ± 1.74 4.36 ± 1.66	4.46 ± 1.81 4.67 ± 1.73	4.16 ± 1.83 3.91 ± 1.78	3.43 ± 1.56 3.31 ± 1.45	3.64 ± 1.45 3.67 ± 1.46	4.02 ± 1.21 3.87 ± 1.22	3.94 ± 1.31 3.98 ± 1.35
		AG Mean ± SD (right/left quadrant)	5.20 ± 1.96 5.11 ± 1.90	5.86 ± 1.22 5.45 ± 1.92	4.98 ± 1.97 5.01 ± 2.01	3.76 ± 1.47 3.83 ± 1.68	4.08 ± 1.53 3.85 ± 1.35	4.50 ± 1.43 4.83 ± 1.60	4.52 ± 1.64 4.42 ± 1.73	3.53 ± 1.69 3.64 ± 1.75	3.52 ± 1.86 3.90 ± 1.72	3.17 ± 1.83 3.11 ± 1.56	2.37 ± 1.45 2.42 ± 1.62	2.50 ± 1.19 2.72 ± 1.51	2.91 ± 1.32 3.01 ± 1.47	2.80 ± 1.29 3.01 ± 1.59
Bhatia et al. (2015) [22] (AG)	India	15-30 years old Mean ± SD (with/without histochemical staining)	4.07 ± 1.08 3.85 ± 0.71 (incisors)		3.8 ± 1.25 3.75 ± 0.95 (canine)	1.92 ± 0.65 2.07 ± 0.73 (premolars)		2.67 ± 0.74 2.8 ± 0.88 (molars)		3.22 ± 0.98 3.12 ± 072 (incisors)		2.45 ± 1.06 2.65 ± 0.94 (canine)	1.5 ± 0.47 1.67 ± 0.31 (premolars)		1.65 ± 0.31 1.92 ± 0.48 (molars)	
		31-45 years old Mean ± SD (with/without histochemical staining)	3.3 ± 0.58 3.62 ± 0.84		2.8 ± 0.71 2.95 ± 0.98	2.07 ± 0.65 2.32 ± 0.75		2.22 ± 0.47 2.42 ± 0.57		3.22 ± 0.73 3.05 ± 0.76		1.8 ± 0.48 1.7 ± 0.42	1.52 ± 0.59 1.47 ± 0.38		1.97 ± 0.65 2.1 ± 0.71	
		46-60 years old Mean ± SD (with/without histochemical staining)	3.75 ± 0.48 3.62 ± 0.47		2.8 ± 0.82 2.75 ± 1.03	2.57 ± 0.61 2.55 ± 0.62		2.47 ± 0.62 2.52 ± 0.67		3.2 ± 0.81 3.12 ± 0.77		2.05 ± 0.89 2.03 ± 0.94	1.77 ± 0.29 1.75 ± 0.31		1.95 ± 0.55 2.07 ± 0.57	
Adesola et al. (2018) [20] (AG)	Nigeria	Mean ± SD (right/left quadrant)	4.86 ± 1.81 4.70 ± 1.64	4.39 ± 1.80 4.39 ± 1.90	3.92 ± 1.55 3.53 ± 1.46	3.28 ± 1.49 3.21 ± 1.43	3.40 ± 1.60 3.23 ± 1.54	3.36 ± 1.71 3.52 ± 1.67	-	3.19 ± 1.15 3.18 ± 1.07	3.35 ± 1.23 3.35 ± 1.23	2.51 ± 1.17 2.51 ± 1.17	2.27 ± 1.17 2.18 ± 1.01	2.78 ± 1.09 2.49 ± 0.93	3.15 ± 1.11 3.16 ± 0.94	-

Ci: central incisor, Li: lateral incisor, Ca: canine, P1: 1st premolar, P2: 2nd premolar, M1: 1st molar, M2: 2nd molar, GM: gingival margin, AG: attached gingiva, KG: keratinized gingiva.
